# A US National Study of Barriers to Science Training Experienced by Undergraduate Students during COVID-19

**DOI:** 10.3390/ijerph19116534

**Published:** 2022-05-27

**Authors:** Sara E. Grineski, Danielle X. Morales, Timothy W. Collins, Shawna Nadybal, Shaylynn Trego

**Affiliations:** 1 Department of Sociology, University of Utah, Salt Lake City, UT 84112, USA; 2 Department of Sociology and Anthropology, University of Texas at El Paso, El Paso, TX 79968, USA; xdeng2@utep.edu; 3 Department of Geography, University of Utah, Salt Lake City, UT 84112, USA; tim.collins@geog.utah.edu (T.W.C.); u1213966@utah.edu (S.N.); satrego@asu.edu (S.T.)

**Keywords:** COVID-19, undergraduate research experiences, motivation, sexual minority status, faculty-mentored research

## Abstract

Undergraduate research is a high-impact practice on college campuses. How the COVID-19 pandemic has affected undergraduate researchers’ progress is poorly understood. We examine how demographics, academic characteristics, research disruptions and faculty mentorship are associated with four barriers to research progress. Data are drawn from a survey of over 1000 undergraduate student researchers across the US. We examine students who actively continued to conduct faculty-mentored research during mid-March/April 2020 (*n* = 485). Using generalized estimating equations that control clustering by institution, we found economic hardship, discomfort teleconferencing, lower quality mentors, sexual minority status and higher grade point averages were associated with motivation problems. Economic hardship, serious illness, Internet connection issues, a lack of face-to-face meetings and lower a frequency of mentor–mentee communication were associated with a time crunch with regard to conducting research. Discomfort teleconferencing, Internet connection issues, a lack of face-to-face meetings and decrease in research workload were associated with task uncertainty. Economic hardship, serious illness and being an engineering major were associated with lacking needed tools for the research. In sum, economic hardship was an important correlate of research barriers, as were communication challenges and sexual minority status. Results can inform practical actions by research program directors and faculty undergraduate research mentors.

## 1. Introduction

As COVID-19 rapidly became a pandemic in spring 2020, college education was immediately and fundamentally altered. Universities transformed their operations. Classes moved online, science lab sessions were cancelled and graduation ceremonies were postponed [1]. Researchers are examining how these changes have impacted student well-being [2,3,4,5], learning and academic engagement [6,7,8], relationships with peers [9] and longer-term outcomes, such as retention and delayed graduation [10,11]. Few studies have examined how COVID-19 has impacted undergraduate research training [12,13,14], and few have focused on initial experiences during the early months of the pandemic [15].

Alongside service learning, learning communities, internships and study abroad programs, faculty-mentored undergraduate research experiences are high-impact practices [16] and critical to a well-rounded college education. Participation in research prepares students for graduate education and careers in research [17,18,19,20,21,22,23,24], while complementing the research workforce composed of graduate students, postdoctoral and faculty researchers. In 2019, 22% of United States (US) college seniors had participated in faculty-mentored research. In some majors, that percentage was substantially higher: 36% of physical science seniors and 46% of biological science seniors had engaged in research [25].

Students accrue many benefits due to participation in faculty-mentored undergraduate research, including improved critical thinking and communication skills, increased interactions with faculty, more extracurricular engagement, enhanced academic achievement and retention and greater persistence to STEM (science, technology, engineering and math) degree completion [26,27,28,29,30]. While majority group students are more likely to participate [25], undergraduate research opportunities are particularly important to the retention and success of students from groups traditionally underrepresented in higher education [17,31,32]. The importance of undergraduate research for student training [16] and its widespread implementation across US universities [25] makes it essential to examine it in the context of COVID-19, especially as undergraduate research training program directors and faculty mentors are planning for future potential disruptions to student research experiences.

How the COVID-19 pandemic has affected undergraduate researchers is poorly understood [13,14,33,34]. To advance knowledge, we developed and administered a survey to over 1000 undergraduate students across the country who were doing or planning to do research in spring and/or summer 2020. Here, we examine the subset of student respondents who were still actively conducting research under the direction of a faculty mentor in mid-March and April 2020. This paper examines how social demographics, academic characteristics, research disruptions and faculty mentorship are associated with four barriers to research progress: experiencing a lack of motivation for conducting research, experiencing a time crunch with regard to conducting research, uncertainty about the next steps in the research project and a lack of access to the tools needed to conduct research. Although research has not established the specific barriers that undergraduate researchers faced during the early months of the COVID-19 pandemic, there is an emerging literature on college students and their transition to online learning, which provides some insight for comprehending the challenges faced by undergraduate researchers. In what follows, we present our conceptual model, which is an input–environment–output (IEO) model [35]. We then review literature relevant to our input and the environment independent variables as well as our dependent variables (outputs).

### 1.1. Conceptual Model

**Figure 1:**
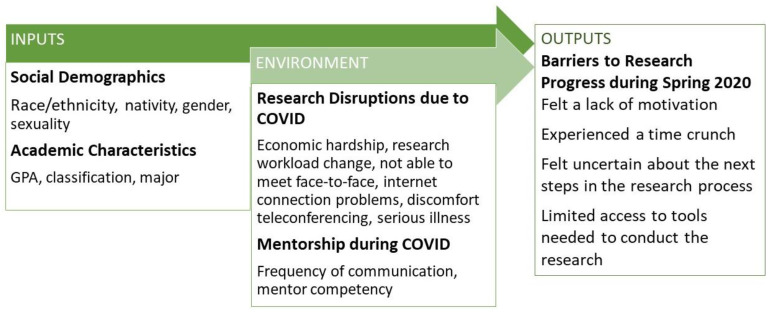
Input–environment–output (IEO) model.

To orient the study, we utilized an input–environment–output (IEO) model [35]. IEO models are used by education researchers to inform the design of statistical analyses in a manner that reduces bias. Specifically, IEO models adjust for the effects of differences in initial student attributes (e.g., sexual minority status) in order to isolate the effects of exposure to specific educational environments (e.g., undergraduate research experiences) while in college on student outcomes. This minimizes the chances of arriving at invalid inferences regarding the influence of an educational environment on student outcomes. The aim of an IEO model is to adjust for potentially biasing covariates in order to accomplish what random assignment enables in controlled experiments by statistical means [36]. “Inputs” is the term used to identify attributes that students bring to the specific educational environment under study. “Environment” represents students’ experiences in the educational environment [35]. “Outputs” refers to the educational outcomes of interest. Figure 1 presents an overview of inputs, environment variables and outputs used in this study. 

#### 1.1.1. Inputs: Social Demographic and Academic Characteristics

Studies of undergraduate research have shown inequalities in experiences and outcomes for students from historically marginalized backgrounds, which we define here to include minority race/ethnicity, foreign-born nativity, woman and non-binary gender identities and sexual minority status. Studies have demonstrated that racial/ethnic minority students tend to gain more from undergraduate research programs than their White peers [23,37,38,39]. However, a few studies find the opposite [30,40]. Only a few studies of undergraduate research have examined the nativity (US-born vs. foreign-born) of the participant [41]. While not a salient factor in the literature on undergraduate research, student nativity is an important input to consider during this pandemic, which the sitting US President blamed on international travelers and immigrants. The pandemic also occurred during a restrictive migration regime, symbolized by border walls, travel bans and hire American policies, which created challenges for foreign-born students [42]. Given these dynamics, it is possible that foreign-born undergraduate researchers might experience greater barriers to research progress during COVID-19 than their US-born counterparts.

In terms of gender identity, differences between women and men have been noted among summer research program participants [37,40]. While sexuality is often less examined in the context of undergraduate research, a national and longitudinal study found that sexual minority STEM students were significantly more likely to participate in undergraduate research than non-sexual minority students [43]. Despite this, sexual minority STEM majors who were similarly positioned to succeed in STEM as their non-sexual minority counterparts were 8% less likely to be retained to the fourth year. This suggests that the retention gap would be even larger if not for disproportionate participation in undergraduate research. How race/ethnicity, nativity, gender and sexuality relate to experiencing barriers to research progress during COVID-19 is not yet known.

Academic characteristics, such as classification, grade point average (GPA) and major, are also influences on undergraduate research participation and experiences. Research experiences are more common in the senior and junior year than they are in the first two years [21]. Limited research has examined how GPA relates to student intentions to persist in their undergraduate research experiences. In one study, students with lower GPAs were more likely to persist in their research experiences because they were worried that they might not have other research opportunities in the future [44]. This may stem from their recognition that students with higher GPAs are more likely to be invited by faculty to engage in research [18]. 

While faculty-mentored undergraduate research has been associated with an increase in GPA [45], nationally, in all majors, less than 8% of first-year students have conducted research with faculty. These percentages grow dramatically by the senior year, with 15% of education majors, 19% of health profession majors, 30% of engineering majors, 30% of social science majors, 36% of physical science/math/computer science majors and 46% of life sciences students having conducted research with faculty [25]. Given the differences in research experience and context, classification, major and GPA are important to consider when studying undergraduate research in COVID-19. We anticipate that first/second year students and those from bench research majors (in which wet laboratory-based work is the norm) will face greater odds of encountering barriers to research progress during COVID-19. We anticipate that those with lower GPAs might face greater odds of encountering research barriers during COVID-19 since they may be struggling with more competing demands in their lives.

#### 1.1.2. Environment in Spring 2020: Research Disruptions and Faculty Mentorship

In addition to the well-established inputs reviewed above, COVID-19 changed the landscape of undergraduate researchers’ lives, their education and their research context. Many college students began to struggle financially due to the pandemic. National data collected in early summer 2020 showed that over 75% of US college students reported some financial hardship [46]. As the spring semester progressed, increasing numbers of undergraduate researchers were unable to meet face to face with their mentors, something that, in pre-COVID-19 times, has been shown to increase the odds of undergraduate students publishing their research [36]. 

In the midst of COVID-19, while nearly all US college students had access to Internet at home (95%), Internet connectivity issues were serious enough to interfere with students’ abilities to attend or participate in their online courses at least “occasionally” for 44% of students, with 16% of students experiencing such problems “often” or “very often” [7]. Internet connectivity problems would also have interrupted remote research activities. Feeling disconnected from others was common in the early weeks and months of COVID-19. While many college students found videoconferencing platforms helpful [47], a minority (8%) lacked familiarity with the technical tools needed for online learning [48]. It is also the case that some students felt uncomfortable videoconferencing in online classes due to embarrassment over their living circumstances or the need to care for dependents during class [49]. Differences in what students see in the backgrounds of their peers’ living spaces via Zoom or other videoconferencing software provide stark reminders of the inequalities between themselves and their classmates that were less obvious in campus classroom environs [50]. 

Getting sick and taking care of ill family members was relatively common during the early weeks of COVID-19 and could have influenced students’ research experiences. One-third of college students had experienced at least one COVID-19-related symptom between February and April 2020, although less than 5% of them were tested [51]. Another national survey of college students documented that 45% of students reported having felt too unwell to participate in online classes at least once [7]. However, it is currently unknown how these potential COVID-19-associated research disruptions (e.g., financial struggles or getting sick) shaped students’ undergraduate research experiences. 

Faculty mentorship is a well-known correlate of successful student experiences in undergraduate research [52]. Higher-quality mentorship from faculty improves student gains [39,41]. Communication is another important element of faculty mentorship of undergraduate researchers. A qualitative analysis of both mentor and mentee perspectives reported that failed mentoring relationships were characterized by poor communication [53]. Working with more competent mentors and communicating with the mentors more frequently may buffer undergraduate researchers from research barriers associated with COVID-19.

#### 1.1.3. Outputs: Barriers to Research Progress

We examined four barriers to research progress: lacking motivation, experiencing a time crunch, lacking necessary tools for research and feeling uncertain about next steps. We selected these four as they were the most commonly reported barriers among those we asked about (which also included concern about losing future opportunities, not receiving a requested letter of reference, and lost opportunity to present at a conference). Motivation is important to examine because it is critical to continued engagement in undergraduate research [54]. Initial research showed that college students struggled with motivation during spring 2020 when their courses switched to online learning [7,48], and that decreases in motivation were associated with lower cognitive engagement in their coursework [6]. Students explained that their motivation suffered as they lacked the structure of regular class routines, were subject to many kinds of distractions and were affected by the chaos of the pandemic [7].

We examine time management challenges as our second research barrier as it is well-established that college students struggled with time management during the pandemic. Even in pre-pandemic times, time management challenges increased student stress [55] and reduced GPAs [56]. COVID-19 has heightened time management challenges for college students [7,57]. Among college students interviewed about their experiences with online learning during the pandemic, time management was one of the key challenges mentioned [58]. 

Our third research barrier is task uncertainty. It is well-known that uncertainty is ubiquitous in the research context. Undergraduate students who are able to navigate the uncertainty inherent in the sciences are more likely to feel that they belong in science and to be retained in their science major [59,60,61]. While uncertainty is integral to the research process, students may abandon research altogether when the uncertainty is too great for them to manage [62]. Thus, uncertainty is an important element to examine in terms of student outcomes during the COVID-19 pandemic. 

We examine lacking access to the tools needed to conduct research as the final barrier because research carried out remotely requires access to specific tools and resources unique to each research project, such as statistical packages and datasets. When courses shifted from in-person courses to remote learning, the success of that transition depended upon student access to computer hardware and software, in addition to Internet connectivity. Research has shown that students without that access struggled with their coursework [48]. One-quarter of college students experienced hardware or software problems severe enough to impact their ability to attend or participate in their online course at least “occasionally” during spring 2020 [7].

### 1.2. Statement of Contribution

Most previous studies have focused on the benefits of undergraduate research experiences [23,26], and fewer studies have examined undergraduates’ challenges while doing research. This is especially relevant during a global health crisis such as the COVID-19 pandemic. The retention of undergraduate students in research now is critical to the development of the future research workforce [21]. Learning more about the correlates of barriers to research progress during COVID-19 will allow undergraduate research program directors and faculty mentors to better support students as this pandemic continues and when similar disruptions happen in the future. This is important because undergraduate research experiences have been shown to be an impactful and successful education model [17,18,19,20,21,22,23], although we do not know how robust this model is in the context of societal disruption. COVID-19 provides an opportunity for those involved to reflect upon and refine undergraduate research training programs to promote resilience moving forward. It is also important to document the challenges undergraduate students have faced during the COVID-19 pandemic in their own right. The pandemic is an important part of the history of higher education and it is critical to understand and document its effects.

## 2. Materials and Methods

### 2.1. Data Collection and Participants

We conducted a survey of undergraduate researchers that asked them about their college experiences, past, current and planned research experiences, mentorship and COVID-19-specific experiences, among other domains. The survey took approximately 30 min to complete. The survey was approved by the Institutional Review Board at the University of Utah. We recruited students through undergraduate research programs at 18 different US universities. We requested program participation through two channels: by emailing program directors affiliated with BUILDing SCHOLARS, which is a National Institutes of Health-funded multi-institutional consortium; and by posting a call for their participation through the Council on Undergraduate Research Listserv.

**Figure 2:**
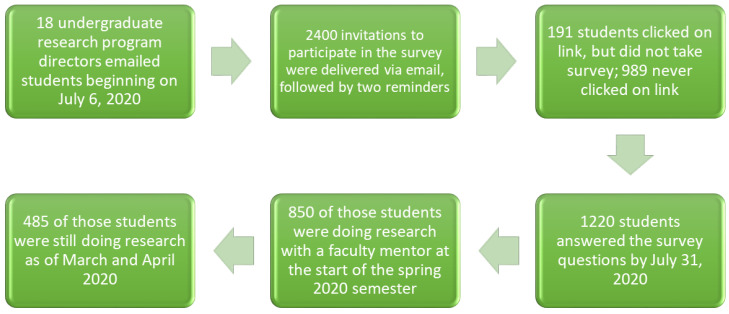
Diagram of the data collection process, including inclusion criteria for this analysis.

The survey was open for 4 weeks during the month of July 2020 and all students received the same invitation and set reminders. We obtained written consent (in a digital format) from participants. All participants received an Amazon gift card as an incentive. Figure 2 displays the data collection process and inclusion criteria for the analyses presented in this paper. Students included in this study were conducting research during mid-March and April 2020 (i.e., during the first months of the COVID-19 pandemic) under the direction of a faculty mentor (*n* = 485).

An additional group of students was conducting research under a faculty mentor at the start of the spring 2020 semester but had stopped conducting research in mid-March, presumably due to the pandemic. They were excluded from these analyses as they were no longer engaged in research during the pandemic. The two groups of students (those who continued doing research and are included in this paper vs. those who stopped doing research and are excluded from these analyses) were quite similar. As per an independent samples t-test, there were no significant differences in terms of GPA, non-White vs. White racial/ethnic status, gender, sexuality or nativity. There were significant differences in terms of several majors. Life sciences majors are overrepresented in the group that stopped doing research (31% of those who continued vs. 54% of those who did not were life sciences majors). “Other major” students are overrepresented in the group that continued doing research (11% vs. 6%) as are social and behavioral sciences majors (15% vs. 7%). Seniors also comprise a larger share of the group that continued to carry out research (51% of those who continued vs. 43% of those who did not).

### 2.2. Independent Variables: Inputs and Environment

Inputs include social demographic (i.e., race/ethnicity, nativity, gender and sexuality) and academic characteristics (i.e., GPA, major and classification). Environment variables in this analysis are students’ COVID-19-specific research disruptions and faculty mentorship variables. Research disruptions include if the student experienced an economic hardship that affected their ability to carry out research, a change in research workload, challenges with not being able to have face-to-face meetings with mentors, Internet connection problems that affected their ability to carry out research, discomfort teleconferencing with mentors and serious illness that affected their ability to carry out research. The environment also includes faculty mentorship variables, i.e., the frequency of communication with the faculty mentor and the student-assessed faculty mentor competency. Table 1 provides more information on these input and environment variables, including the survey question used and how the variable is coded in our statistical models. 

Table 2 presents descriptive statistics. Over half of the students were non-White (65.0%), women (58.1%) and seniors (50.7%). Just under one-fifth were sexual minority (18.8%). The average major GPA of the participants was 3.74. One-quarter were engineering majors (24.5%), 14.8% were social/behavioral sciences majors, 12.7% were math/computer science/physical science majors and 6.3% were in a health professional major. They rated their faculty mentor 4.07, on average, on a five-point scale. In terms of research disruptions, 28.7% reported that economic hardships disrupted their research experience, 42.6% reported the same for internet connection issues. Approximately one-fifth (17.5%) were uncomfortable teleconferencing and 12.0% were disrupted by serious illness.

### 2.3. Dependent Variables: Outputs

Our dependent variables represent four common barriers to research progress that students experienced during spring 2020 (see “outputs” in Table 2). We asked students to indicate whether or not they had experienced “feeling a lack of motivation”, “a time crunch”, “feeling uncertain about the next steps in the research process” and “limited access to the tools needed to conduct the research” caused by COVID-19 that could have affected their ability to conduct research during the time period of mid-March–April 2020. Each of these variables is coded 1 = Yes and 0 = No and analyzed without transformation. Among our sample, as shown in Table 2, 68.4% “felt a lack of motivation”, 56.5% reported “experiencing a time crunch”, 63.7% “felt uncertain about the next steps” and 58.9% had “limited access to the tools needed”.

### 2.4. Statistical Methods

We began the analysis by conducting multiple imputation (MI). Multiple imputation is a well-established practice for dealing with missing data. When researchers only include cases without any missing data, this can introduce bias into the results. When using multiple variables in a model, missing values across all the variables can substantially reduce the sample size, and therefore statistical power and precision (even if the complete case analysis does not introduce bias) [64].

Our MI approach consisted of creating multiple sets of values for missing observations using a regression-based approach [65]. This enabled us to avoid the bias that can occur when missing values are not missing completely at random [65]. The imputation procedure fully accounts for uncertainty in predicting the missing values by injecting appropriate variability into the multiple imputed values [64]. In IBM SPSS Statistics 25, we created 20 multiply imputed datasets, each separated by 200 iterations, with the imputed values at each 200th iteration saved as an imputed dataset [65]. When analyzing multiply imputed data in statistical models, the standard errors are calculated to take into account the variability in results across the imputed datasets and thus the uncertainty associated with the missing values [66]. Because the models average over the distribution of the missing data given the observed data, valid inferences are obtained [64].

We analyzed the multiply imputed data using multivariable generalized estimating equations (GEEs) predicting each of our four dichotomous dependent variables. GEEs build from the generalized linear model and provide a general method for the analyses of clustered continuous, ordinal, dichotomous, polychotomous and event-count response variables. GEEs relax several assumptions of traditional regression models [67]. GEEs assume that observations from within a cluster are correlated, whereas observations from different clusters are independent [67]. GEE models utilize an intracluster dependency correlation matrix that we specified as exchangeable, which assumes constant intracluster dependency [68]. GEEs are able to estimate unbiased population-averaged (i.e., marginal) regression coefficients, even with misspecification of the correlation structure when using a robust variance estimator [69,70], as we do here. 

We used the student’s home institution to define clusters (*n* = 49). While we recruited students through 18 research programs, some of these programs serve students from across the US during the summer. This led to the inclusion of students from 49 different home universities in the analyses. The number of students in each cluster ranged from 1 to 98 students. By defining home institution as the cluster variable, we are able to control home institutional effects as a nuisance parameter. Because our dependent variables are dichotomous, we employed GEEs which use a binomial distribution with a logarithmic link function. Model results are not affected by multicollinearity.

The quasi-likelihood estimating equations have the general form

∑i(∂μi∂β)′v(μi)−1[yi−μi(β)]=0

where $\mu_{i}=g^{-1}\left(X^{\prime }\beta \right)$ is the link function with *g* = *logarithmic*, the distribution of $y_{i}$ is negative binomial and the GEE estimator ($\hat{\beta }$) is the solution to these equations. The resulting covariance of the GEE is given by

$$V_{G,n}=n{\left[\underset{i}{\sum }D_{i}^{\prime }V_{i}^{-1}D_{i}\right]}^{-1}\left[\underset{i}{\sum }D_{i}^{\prime }V_{i}^{-1}cov\left(Y_{i}\right)V_{i}^{-1}D_{i}\right]{\left[\underset{i}{\sum }D_{i}^{\prime }V_{i}^{-1}D_{i}\right]}^{-1}$$

and is assumed to be compound symmetric. For more information about GEEs, see Zorn [71].

## 3. Results

Table 3A depicts results from the GEEs predicting the odds of students’ lacking motivation to complete their research due to COVID-19. In terms of the inputs, sexual minority students were twice as likely to lack motivation than non-sexual minority students (*p* < 0.05), and a one-point increase in GPA was associated with 1.95 times greater likelihood of lacking motivation (*p* < 0.05). Several environment variables were statistically significant. Students who were experiencing economic hardship were 1.6 times more likely to lack motivation (*p* < 0.05). Those who felt uncomfortable teleconferencing were 2.3 times more likely to lack motivation (*p* < 0.01). Rating their mentor one point higher on the mentor competency scale was associated with a 27% reduction in the odds of a student lacking motivation (*p* < 0.05). 

Table 3B shows results from the model predicting the odds of students experiencing a time crunch with their research due to COVID-19. None of the input variables were statistically significant. Four environment variables were significantly associated with a time crunch. The challenge of not meeting face to face was associated with a 2.1 times increase in the odds of a time crunch (*p* < 0.05). Struggling with Internet access was associated with 1.5 times greater odds of a time crunch (*p* < 0.01). If the student or the student’s family got seriously ill in spring 2020, the student was 1.2 times more likely to report a time crunch (*p* < 0.05). A one unit increase in the communication frequency variable (scaled such that an increase corresponds to less frequent communication with the faculty mentor was associated with a 1.2 times increase in the odds of a time crunch (*p* < 0.01). 

Results for the model predicting the odds of students’ feeling uncertain about the next steps in their research projects (i.e., task uncertainty) due to COVID-19 are shown in Table 4C. In terms of inputs, no findings were significant. In terms of environment variables, an increase in research workload was associated with a 21% decrease in the odds of task uncertainty (*p* < 0.001). The challenges of not meeting face to face and Internet problems increased the odds of task uncertainty by 1.7 and 1.6 times, respectively (*p* < 0.05). Feeling uncomfortable with teleconferencing was associated with a 3.0 times increase in the odds of task uncertainty (*p* < 0.001).

Table 4D presents results from the GEE predicting the odds of a student lacking the tools they needed to do research due to COVID-19. In terms of inputs, engineering majors were 2.1 times more likely to lack tools compared to life sciences majors (*p* < 0.05) and social and behavioral sciences majors were 57% less likely than life sciences majors to lack tools (*p* < 0.001). Among the environment variables, experiencing economic hardship increased the odds of lacking tools by 2.1 times (*p* < 0.001). Reporting serious illness (on behalf of the student or their family) increased the odds of lacking tools by 1.8 times (*p* < 0.05). In sum, Table 5 reviews the significant predictors (*p* < 0.05) for each barrier to research progress.

We explored some sensitivity analyses as part of the modeling. We ran the models with the racial/ethnic groups disaggregated into four groups (i.e., Black, Asian, Latinx and other) with non-Hispanic White as the reference; there were no significant differences, so we grouped them together to increase statistical power. We also tried including first-generation student status in the model, but that covariate did not approach statistical significance in any of the models, so we did not include it in the final models. We also explored international student status in place of foreign-birth, but the results for that variable were not statistically significant and were therefore not included.

## 4. Discussion

Overall, economic hardship was an important correlate of research barriers, as were communication issues (e.g., teleconferencing, Internet problems, no face-to-face meetings). Faculty mentorship was less related to the barriers, although working with high-quality mentors reduced students’ motivation challenges. There were disciplinary differences regarding which students lacked tools.

Input variables were less closely related to the barriers than the environment variables, suggesting that the barriers were more closely related to changes in COVID-19-associated research environments than students’ social demographic and academic characteristic inputs. Specifically, there were no racial/ethnic, nativity or gender differences in the experience of any of the barriers to research progress during COVID-19 (Table 3 and Table 4). Sexual minority students were twice as likely to lack motivation as compared to their non-sexual minority counterparts (Table 3A), but they were not significantly more likely to experience a time crunch, be uncertain about next steps or to lack tools (Table 3B and Table 4). The motivation disparity with regard to sexual minority status is concerning for several reasons. Sexual minorities are already underrepresented in STEM [72,73]. They are less likely to declare a STEM major [73] and less likely to be retained in a STEM major [72], even though they participate in undergraduate research at higher levels than non-sexual minority students [43]. This suggests there are likely barriers related to the mentoring of sexual minority students in their research training environments [43]. Other research on sexual minority students during COVID-19 has reported that, while they might be “out” among their college campus community, they may not be “out” at home or they may have returned home to hostile or unsupportive families or communities [74]. We believe that this may be linked to research motivation as other research has identified social support as an important correlate of academic motivation [75].

With regard to academic characteristics, we found that students with higher GPAs were more likely to lack motivation (Table 3A). Interestingly, pre-COVID-19, students with higher GPAs were more likely to have quit their research placement than those with lower GPAs, primarily because they did not enjoy their everyday research tasks [44]. Assuming those results are generalizable, they imply that our students with higher GPAs may have been more likely to dislike their research tasks before COVID-19, suggesting that they might have been less motivated with the onset of COVID-19 to continue those tasks. While speculative, conversations between the authors and several research program directors also suggested that high achieving students had higher expectations for their research experiences, which were not well-met with online research, resulting in lower levels of motivation during the pandemic.

While few disciplinary variables were significant, we did find that engineering students lacked tools, while social sciences students did not, relative to life sciences students (Table 4D). It is important to note that the comparison group of life sciences students was overrepresented among the students who had discontinued research by mid-March 2020, suggesting that they too may have faced this barrier (although this was not tested directly). It is likely that it was difficult or impossible to replace the technology needed to conduct engineering research (and potentially life sciences research as well) in an online framework without considerable lead-time for planning.

Research disruptions (environment variables) were strongly linked to the barriers, controlling for the inputs. Experiencing research-disrupting economic hardships increased the odds of lacking motivation (Table 3A) and lacking tools (Table 4D). Students without economic hardship likely already had, or could purchase, a sufficiently powerful computer to conduct analyses, and they could also have more easily purchased any needed software packages to conduct their research. Students who got seriously ill and/or had a family member become ill in the early days of the pandemic were more likely to face the barriers of a time crunch (Table 3B) and a lack of tools (Table 4D). While only 12% of students suffered from this experience, when they did, it appears as if it reduced the time that they could devote to research. Illness also contributed to students lacking access to the tools needed to conduct research (Table 4D). In the authors’ cases, we know that the process of setting undergraduates up to work remotely through an online server was time-consuming and involved substantial troubleshooting, which may have been nearly impossible if a student was seriously ill or busy caring for ill family members. 

Interestingly, an increase in research workload during COVID-19 was associated with a significant decrease in task uncertainty (Table 4C). The average student reported a slight decrease in workload relative to the pre-COVID-19 baseline. The average student in our sample was working approximately 10 h per week on research as of April 2020, yet 25% were still working over 15 h/week. While research workload is a rarely examined factor in the undergraduate research literature, feeling overworked/undervalued was not a reason given frequently by undergraduate researchers who had quit their research experience pre-COVID-19 [44]. We probed this finding by adding a quadratic term (i.e., workload*workload, b = 0.097, *p* < 0.004) to the GEE. The graphed result (not shown) showed that the protective (negative) effect of workload on task uncertainty was initially steep (i.e., when workload values were between 1 and 3 meaning that the student was working less than usual), then the negative effect tapered off at mid-levels of increased workload (i.e., when workload values were between 4 and 5 meaning the student was working the same or only slightly more) and the effect became positive (i.e., a risk factor for uncertainty) at the highest level of workload (i.e., when workload values were between 6 and 7 meaning that the student was working moderately more or much more). The finding shows that task uncertainty was at its lowest when students were spending similar amounts of time on their research during COVID-19 as they did pre-pandemic. 

The challenges of not meeting face to face and unreliable Internet access were linked to increased odds of task uncertainty (Table 4C) and of a time crunch (Table 3B). Interestingly, these variables were not associated with lack of motivation (Table 3A). In terms of face-to-face meetings, students who could not meet face to face with their mentors or research teams struggled with finding time for research (time crunch, Table 3B) and with knowing what to do (task uncertainty, Table 4C). In-person communication is an important part of the mentor–mentee relationship, which the pandemic fundamentally disrupted (e.g., 89.9% of students in this sample reported this problem). After research mentoring became virtual due to COVID-19 at one US university, undergraduates reported feeling lost and missing opportunities for informal communication [15]. Internet problems contributed to these two barriers as well. Research on 23 remote 2020 summer research programs identified technology issues as one of the challenges that limited participants’ experiences. Some students did not have suitable Internet connections, access to computers with sufficient computing capacity or credentialing to allow access to essential software [13]. One can imagine students struggling with the time pressures it takes to troubleshoot Internet issues or set up new services. Problems with Internet service could also leave them uncertain about next steps, as they likely missed out on timely communications from their mentors or research team (via email or video chat). 

Feeling uncomfortable with teleconferencing was also associated with task uncertainty (Table 4C) in addition to lacking motivation (Table 3A). While discomfort teleconferencing affected just under 20% of our sample, the effect sizes in the GEEs for this variable are notable. Discomfort with teleconferencing increased the odds of task uncertainty and lacking motivation by 3.0 and 2.3 times, respectively. Discomfort with teleconferencing could stem from a variety of sources, including technical difficulties due to Internet, hardware or software issues, competing caretaking demands and/or embarrassment about one’s surroundings [49,50]. In our dataset, disadvantaged students were more likely to report feeling uncomfortable teleconferencing. For example (table not shown), 21% of sexual minority students reported feeling uncomfortable while 16% of non-sexual minority students reported the same. The percentages were 20% vs. 15% for women vs. men and 18% vs. 15% for non-White vs. White students.

Mentorship was a significant correlate of two barriers. More frequent communication was protective against students reporting a time crunch (Table 3B). Students who communicated frequently were likely to have opportunities to ask questions and receive instructions, making their research time more efficient. In terms of how much communication students were engaging in, the average student reported a communication frequency score of 3.3 on a scale of 1 to 6, with 3 corresponding to “once per week” and 4 to “several times per month.” Indeed, this was less than what was typical before the pandemic. Pre-COVID-19, 81.5% of students communicated with their mentor at least once per week with 55.7% communicating at least several times per week (table not shown). During COVID-19, only 47.5% reported communicating with their mentor at least once per week and 19.1% communicated at least several times per week. At the other end of the spectrum, 28.9% reported speaking to their mentor once per month or less during the COVID-19 (table not shown). Pre-COVID-19, only 6.4% reported speaking with their mentor once per month or less. 

It was notable that more competent mentors were a significant factor in relieving motivation problems (Table 3A). Identifying factors that improved student motivation under COVID-19 conditions is important since motivation was the first-ranked problem affecting college students in spring 2020 [7,48] and it affected 68% of undergraduate researchers here. More competent mentors have been linked to other important outcomes such as greater gains in science identity, research skills and personal skills [39]. During COVID-19, faculty mentor competency has been linked to graduate school intentions. Specifically, undergraduate researchers who had less competent faculty mentors (vs. more competent mentors) were 3.6% less motivated by COVID-19 to pursue a graduate degree in science [14].

### Limitations

Selection bias could have impacted our findings. We do not know if students with more severe research challenges were more or less likely to participate. It may have been that students who were struggling the most were less likely to click on the link to take the survey due to competing demands on their time. Or, the survey may have attracted those students, as they may have felt they had much to contribute to the study. Overall, students whose research experiences were cancelled may have been less likely to participate in the survey. However, that would not influence this analysis since we only examined students who were conducting research through the entire spring 2020 semester. We are limited by only having dichotomous measures of the four barriers, which means we do not know the degree to which students may have struggled in each area. We also do not have more information about *why* they felt (un)comfortable teleconferencing, which would have furthered our ability to interpret that finding. 

We asked students to recall their experiences during March–April 2020 in July 2020, which could introduce recall bias, although we conducted the survey as soon as possible. It is also the case that many students had already stopped doing research by mid-March 2020, possibly due to overwhelming challenges which are not captured in this study. For example, we saw that life sciences students were overrepresented among students who had stopped doing research by mid-March, potentially because it was too challenging to conduct life sciences research online without time to prepare. However, we could not include those students in our GEEs since the barrier items pertained only to students with active research experiences during mid-March/April 2020. Additionally, this paper is focused on COVID-19-related barriers to research progress, but there are likely ways in which COVID-19 was beneficial to students’ research progress, e.g., increasing their motivation to conduct real world research or their desire to go to medical school [76]. While those outcomes are important, they are beyond the scope of this paper.

## 5. Conclusions

Our study findings have practical implications that are relevant to research program directors as well as faculty mentors to undergraduate researchers. These are summarized in Table 6. These implications may also be relevant to anyone working with college students during COVID-19, since challenges affecting undergraduate researchers seem to reflect broader challenges on college campuses. 

While motivation is complex and influenced by a variety of factors, faculty mentors and research program directors can influence student motivation. Others engaged in studying emergency online learning during COVID-19 have recommended that professors prompt students to write out reasons why school is important for them or send out small announcements of encouragement, as two simple ways to boost student motivation. These approaches can easily be adapted to a research context, wherein trainees can be prompted to reflect on why they are involved in research to begin with and the value of their research projects. Mentors can also send encouraging emails to their student mentees regularly. As mentor training has been shown to improve mentor competency [77] and more competent mentors relieved COVID-19-related motivation issues here, mentor training, e.g., the Entering Mentoring curriculum, see [78], should continue to become an important part of faculty development among scholars working with undergraduate researchers.

We found sexual minority students to be affected to a greater degree by motivational barriers, which raises the concern that COVID-19 might push even more sexual minority students away from STEM. This implies that program directors and faculty mentors should implement strategies to retain these students now. To support sexual minority students, faculty and research program directors can use their own position of privilege to be allies [79]. They can engage in behaviors that outwardly support diversity by openly expressing their ally identities and by role modeling their support for all diverse groups. Mentors can emphatically state out loud their genuine support for minority groups and diversity-supportive causes and share information about diversity-related events on campus with their research team. Upon returning to campus post-COVID-19, they can post-ally/diversity-supportive stickers in their offices. Faculty who express their ally identities in these ways convey their desire to create safe spaces for students [79]. When faculty engage in and promote inclusive behaviors, they encourage students with hidden differences to feel comfortable disclosing their identities within the research team, which can engender positive changes including an improved commitment to the research team [80].

In terms of accessing tools needed to conduct research remotely, economic and disciplinary factors were the most salient correlates in our analysis. This suggests that students might need financial assistance to acquire the tools needed to do remote research, and that research programs serving engineering students must be sensitive to their particular research training circumstances. Many engineering faculty mentors and program directors have creatively sought ways to facilitate authentic remote research experiences for their undergraduate trainees. Some have sent lab kits home with students and others have designed virtual reality experiments. Access to these sorts of “research at home” opportunities will need to expand as long as students are unable to fully return to campus life. Even after COVID-19, the growth in remote research opportunities spurred by the pandemic might create new participation opportunities for students who had previously been excluded from research (e.g., part-time students that also work full-time, home-bound students, students that care for dependents). Program directors and mentors should be attuned to these opportunities. 

To address the barrier of task uncertainty, interventions are needed to address mentor–mentee communication challenges, since those are at the root of task uncertainty based on our model results. Opening lines of communication regarding teleconferencing is an important first step here. This might help mentors assess if this is a problem for their mentees and how they might address it, given that it is strongly linked to task uncertainty (as well as motivation).

To address the barrier of students feeling a time crunch with their research, both economic and communication challenges must be addressed. On the communication side, there are techniques that mentors can use to help mentees manage their time. Ho [81] reviewed literature on time management and suggests that mentors and mentees can devise a week-by-week schedule of activities for the project, with due dates, and then follow it. Mentors can build ‘catching up time’ into the schedule to allow for slight delays; divide the project up into small manageable units, which can make the project feel more attainable to the student; and finally, ask for regular submission of written work, as opposed to waiting until the end of the project [81]. Despite being written about years before COVID-19, these strategies are easily adaptable to the pandemic context.

In sum, the barriers to research progress identified in this study were more closely related to the changing circumstances induced by the pandemic than to student social demographics or academic characteristics. This provides opportunities for interventions to change the context so that students are less likely to experience these barriers moving forward under COVID-19 conditions and in the future.

## Figures and Tables

**Table 1:** Descriptions of input and environment variables used in the analysis.

Variable Name	Survey Question	Coding
INPUTS		
*Social Demographic Characteristics*		
Non-White	What is your race/ethnicity? (Pick just one)	1 = non-White (combines Hispanic, Black non-Hispanic, Asian non-Hispanic and other, which included Native American, Native Hawaiian, Pacific Islander, Multiracial and Other), 0 = White and non-Hispanic
Non-US nativity	Were you born in the US?	1 = Other country, 0 = US
Gender	What is your gender/gender identity?	3 categories: each coded 1 = yes, 0 = no. Beyond binary (i.e., trans man, trans woman, genderqueer/gender non-conforming, Other); woman; man (reference)
Sexual minority status	What is your sexual orientation?	1 = Yes (gay, bisexual, lesbian, pansexual, asexual or other); 0 = no (non-sexual minority)
*Academic Characteristics*		
Major GPA	Based on the 4-point scale, what was your GPA in spring 2020 in your major?	Continuous
Classification	Based on the credit hours you have taken, what was your class level in spring 2020?	3 categories, each coded 1 = yes, 0 = no. Senior; junior; first or second year (reference)
Major	Please select your major from the list below. The list includes 90 majors taken from the Higher Education Research Institute’s Freshman Survey.	6 categories, each coded 1 = yes, 0 = no. Engineering; health professions; social and behavioral sciences; math/computer science/physical sciences; other majors; life sciences (reference)
ENVIRONMENT		
*Research Disruptions*		
Economic hardship	Did the student experience economic hardships caused by COVID-19 that affected their ability to conduct research during the time period of mid-March-April 2020?	1 = yes, 0 = no
Research workload change	How much did your spring 2020 research workload change as a result of COVID-19?	1 (working much less) to 7 (working much more)
Could not meet face to face	Did the student experience the following challenge, caused by COVID-19 that affected their ability to conduct research during the time period of mid-March-April 2020?	1 = yes, 0 = no
Internet connection problems	Did the student experience the following challenge, caused by COVID-19 that affected their ability to conduct research during the time period of mid-March-April 2020?	1 = yes, 0 = no
Felt uncomfortable teleconferencing	Did the student experience the following challenge, caused by COVID-19 that affected their ability to conduct research during the time period of mid-March-April 2020?	1 = yes, 0 = no
Student or family member got seriously ill in spring 2020	Did the student experience the following challenge, caused by COVID-19 that affected their ability to conduct research during the time period of mid-March-April 2020?	1 = yes, 0 = no
*Mentorship*		
Frequency of communication with mentor during-COVID-10	How often did you usually communicate with your primary faculty mentor during COVID-19?	1 = daily to 6 = less than once a month
Faculty mentor competency	The student answered 26 Likert items about their mentor’s competency, which are averaged to create the score [63].	1 (low competency) to 5 (high competency)

**Table 2:** Descriptive statistics.

	N	Min.	Max.	Mean	Std. Dev.	Yes	No	% Missing
INPUTS								
Non-White	429					279	150	11.55%
Non-US Nativity	431							11.13%
Woman (ref: man)	430					250	180	11.34%
Beyond binary (ref: man)	430					9	421	11.34%
Sexual minority status	421					79	342	13.20%
Major GPA	462	2.00	4.00	3.74	0.36			4.74%
Senior (ref: first/second year)	473					240	233	2.47%
Junior (ref: first/second year)	473					164	309	2.47%
ENVIRONMENT								
Economic hardship	474					136	338	2.27%
Research workload changed due to COVID-19	474	1	7	2.98	1.76			2.27%
Could not meet face to face	474					426	48	2.27%
Internet connection problems	474					202	272	2.27%
Felt uncomfortable teleconferencing	474					83	391	2.27%
Student or family member got seriously ill in spring 2020	474					57	417	2.27%
Frequency of communication with mentor during COVID-19	455	1	6	3.32	1.241			6.19%
Faculty mentor competency	441	1.27	5.00	4.07	0.75			9.07%
Engineering (ref: life sci)	474					116	358	2.27%
Health prof. (ref: life sci)	474					30	444	2.27%
Soc/Behav. sci. (ref: life sci)	474					70	404	2.27%
Math/CS/Phys (ref: life sci)	474					60	414	2.27%
Other major (ref: life sci)	474					51	423	2.27%
OUTPUTS								
Felt a lack of motivation	474					324	150	2.27%
Experienced time crunch	474					268	206	2.27%
Felt uncertain about next steps	474					302	172	2.27%
Limited access to tools	474					279	195	2.27%

**Table 3:** GEE models predicting the odds of undergraduate researchers (A) lacking motivation for research and (B) experiencing a time crunch.

	A				B			
	Exp(B)	Lower 95% CI	Upper 95% CI	*p*	Exp(B)	Lower 95% CI	Upper 95% CI	*p*
Intercept	0.227	0.017	3.105	0.267	0.179	0.007	4.393	0.292
INPUTS								
Non-White	0.935	0.610	1.435	0.758	1.531	0.972	2.411	0.066
Non-US Nativity	1.316	0.814	2.128	0.262	1.055	0.651	1.713	0.827
Man (ref)								
Woman	1.394	0.964	2.016	0.078	1.124	0.755	1.672	0.566
Sexual minority status	**1.965**	**1.065**	**3.626**	**0.031**	1.25	0.747	2.092	0.395
Beyond binary	0.795	0.242	2.609	0.702	1.359	0.414	4.459	0.611
Major GPA	**1.951**	**1.051**	**3.622**	**0.034**	0.874	0.365	2.092	0.762
First/second year (ref)								
Senior	1.071	0.589	1.948	0.822	1.144	0.742	1.765	0.541
Junior	1.212	0.616	2.385	0.578	0.953	0.511	1.779	0.881
Life sciences major (ref)								
Engineering	1.205	0.642	2.261	0.561	1.311	0.806	2.134	0.276
Health prof.	0.7	0.341	1.433	0.329	1.051	0.469	2.356	0.905
Soc/Behav. sci.	1.18	0.658	2.117	0.578	1.686	0.611	4.651	0.313
Math/CS/Phys. sci.	1.299	0.733	2.303	0.37	1.366	0.654	2.852	0.406
Other major	0.942	0.523	1.697	0.842	0.805	0.426	1.520	0.503
ENVIRONMENT								
Economic hardship	**1.583**	**1.033**	**2.425**	**0.035**	**2.172**	**1.323**	**3.564**	**0.002**
Research workload	1.029	0.940	1.127	0.536	1.087	0.963	1.228	0.18
Could not meet face to face in spring 2020	1.694	0.850	3.374	0.134	**2.088**	**1.121**	**3.892**	**0.02**
Internet connection problems in 2020	1.039	0.682	1.582	0.859	**1.5**	**1.106**	**2.034**	**0.009**
Felt uncomfortable teleconferencing	**2.227**	**1.349**	**3.680**	**0.002**	1.611	0.804	3.228	0.179
Student or family member got seriously ill in spring 2020	1.14	0.662	1.964	0.637	2.188	1.105	4.332	**0.025**
Frequency of communication with mentor during COVID-19	0.933	0.754	1.154	0.523	**1.173**	**1.055**	**1.305**	**0.003**
Faculty mentor competency	**0.727**	**0.548**	**0.966**	**0.028**	0.972	0.77	1.228	0.813

Note: Models use a binomial distribution with log link function and an exchangeable correlation matrix. They adjust for clustering by home university. There are 49 clusters, with 1–98 students/cluster. Bold font denotes findings that are significant at *p* < 0.05.

**Table 4:** GEE models predicting the odds of undergraduate researchers (C) experiencing research task uncertainty and (D) lacking the tools needed to do research.

	C				D			
	Exp(B)	Lower 95% CI	Upper 95% CI	*p*	Exp(B)	Lower 95% CI	Upper 95% CI	*p*
Intercept	0.355	0.024	5.270	0.452	1.064	0.112	10.095	0.957
INPUTS								
Non-White	1.198	0.675	2.125	0.537	1.273	0.785	2.063	0.327
Non-US Nativity	0.92	0.626	1.351	0.669	0.741	0.512	1.073	0.112
Man (ref)								
Woman	1.364	0.777	2.394	0.279	1.055	0.607	1.835	0.849
Beyond binary	1.349	0.440	4.137	0.598	0.77	0.264	2.246	0.631
Sexual minority status	1.014	0.533	1.929	0.967	1.288	0.788	2.106	0.311
Major GPA	1.858	0.984	3.508	0.056	1.362	0.830	2.234	0.221
First/second year (ref)								
Senior	0.55	0.282	1.073	0.079	0.683	0.376	1.241	0.211
Junior	0.775	0.393	1.530	0.462	0.622	0.297	1.305	0.209
Life sciences major (ref)								
Engineering	0.873	0.573	1.327	0.524	2.099	1.058	4.166	**0.034**
Health prof.	0.907	0.425	1.937	0.802	0.575	0.268	1.234	0.155
Soc/Behav. sci.	1.313	0.804	2.145	0.276	0.426	0.258	0.702	**0.001**
Math/CS/Phys. sci.	0.741	0.467	1.175	0.203	0.751	0.453	1.246	0.268
Other major	1.339	0.713	2.514	0.364	0.573	0.268	1.221	0.149
ENVIRONMENT								
Economic hardship	1.316	0.687	2.519	0.408	2.081	1.344	3.219	**0.001**
Research workload	0.791	0.692	0.904	**0.001**	0.941	0.807	1.096	0.433
Could not meet face to face in spring 2020	1.718	1.005	2.936	**0.048**	1.188	0.598	2.363	0.623
Internet connection problems in 2020	1.625	1.113	2.373	**0.012**	1.061	0.658	1.709	0.808
Felt uncomfortable teleconferencing	3.013	1.726	5.259	**<0.001**	1.127	0.652	1.946	0.669
Student or family member got seriously ill in spring 2020	1.28	0.638	2.568	0.488	1.807	1.096	2.980	**0.02**
Frequency of communication with mentor during COVID-19	1.06	0.929	1.209	0.388	0.989	0.835	1.171	0.899
Faculty mentor competency	0.783	0.593	1.034	0.084	0.822	0.610	1.107	0.197

Note: Models use a binomial distribution with log link function and an exchangeable correlation matrix. They adjust for clustering by home university. There are 49 clusters, with 1–98 students/cluster. Bold font denotes findings that are significant at *p* < 0.05.

**Table 5:** Summary of significant findings for input and environment variables (from Table 4 and Table 5).

	Research Barriers			
	Lacking Motivation	Time Crunch	Task Uncertainty	Lacking Tools
**Inputs (*p* < 0.05)**	○ Sexual minority ○ Higher GPA	○ None	○ None	○ Engineering major ○ Not social/behavioral major
**Environment Variables (*p* < 0.05)**	○ Economic hardship ○ Discomfort teleconferencing ○ More competent mentor	○ Economic hardship ○ Serious illness ○ Internet problems ○ No face-to-face meetings	○ Internet problems ○ Discomfort teleconferencing ○ No face-to-face meetings ○ Workload increase	○ Economic hardship ○ Serious illness

**Table 6:** Summary of practical implications by each barrier to research progress.

	Research Barriers			
	Lacking Motivation	Time Crunch	Task Uncertainty	Lacking Tools
**Practical** **Implications**	○ Guided reflection on the importance of research ○ Encouraging emails ○ Faculty mentor training ○ Modelling ally behaviors	○ Teaching research time management, including frequent due dates	○ Open lines of communication ○ Conversations about teleconferencing	○ Financial assistance ○ Creative remote options for engineers

## Data Availability

Data are available from the authors upon request.
